# Crystal structures of Sr(ClO_4_)_2_·3H_2_O, Sr(ClO_4_)_2_·4H_2_O and Sr(ClO_4_)_2_·9H_2_O

**DOI:** 10.1107/S1600536814024726

**Published:** 2014-11-15

**Authors:** Erik Hennings, Horst Schmidt, Wolfgang Voigt

**Affiliations:** a TU Bergakademie Freiberg, Institute of Inorganic Chemistry, Leipziger Strasse 29, D-09596 Freiberg, Germany

**Keywords:** crystal structure, low-temperature salt hydrates, perchlorate hydrates, strontium salts

## Abstract

The title compounds, strontium perchlorate trihydrate {di-μ-aqua-aquadi-μ-perchlorato-strontium, [Sr(ClO_4_)_2_(H_2_O)_3_]_*n*_}, strontium perchlorate tetra­hydrate {di-μ-aqua-bis­(tri­aqua­diperchloratostrontium), [Sr_2_(ClO_4_)_4_(H_2_O)_8_]} and strontium perchlorate nona­hydrate {hepta­aqua­diperchloratostrontium dihydrate, [Sr(ClO_4_)_2_(H_2_O)_7_]·2H_2_O}, were crystallized at low temperatures according to the solid–liquid phase diagram. The structures of the tri- and tetra­hydrate consist of Sr^2+^ cations coordinated by five water mol­ecules and four O atoms of four perchlorate tetra­hedra in a distorted tricapped trigonal–prismatic coordination mode. The asymmetric unit of the trihydrate contains two formula units. Two [SrO_9_] polyhedra in the trihydrate are connected by sharing water mol­ecules and thus forming chains parallel to [100]. In the tetra­hydrate, dimers of two [SrO_9_] polyhedra connected by two sharing water mol­ecules are formed. The structure of the nona­hydrate contains one Sr^2+^ cation coordinated by seven water mol­ecules and by two O atoms of two perchlorate tetra­hedra (point group symmetry ..*m*), forming a tricapped trigonal prism (point group symmetry *m*2*m*). The structure contains additional non-coordinating water mol­ecules, which are located on twofold rotation axes. O—H⋯O hydrogen bonds between the water mol­ecules as donor and ClO_4_ tetra­hedra and water mol­ecules as acceptor groups lead to the formation of a three-dimensional network in each of the three structures.

## Abstract 2

The crystal structures of the tri-, tetra- and nona­hydrate phases of Sr(ClO_4_)_2_ consist of Sr^2+^ ions coordinated by nine oxygen atoms from water mol­ecules and perchlorate tetra­hedra. O—H⋯O hydrogen bonds between water mol­ecules and ClO_4_ units lead to the formation of a three-dimensional network in each of the structures.

## Chemical context   

The amount of research into perchlorates has increased considerably in the last few years, beginning with the Phoenix Mars mission (Kim *et al.*, 2013 ▶; Kerr, 2013 ▶; Chevrier *et al.*, 2009 ▶; Quinn *et al.*, 2013 ▶; Davila *et al.*, 2013 ▶; Gough *et al.*, 2011 ▶; Navarro-González & McKay, 2011 ▶; Robertson & Bish, 2011 ▶; Schuttlefield *et al.*, 2011 ▶; Navarro-González *et al.*, 2010 ▶; Marion *et al.*, 2010 ▶; Hecht *et al.*, 2009 ▶). Important perchlorate salts in the martian regolith are Mg and Ca perchlorates. It seemed worthwhile to complete the chemical systematics in this series of alkaline-earth perchlorates. The solubility diagram of strontium perchlorate has been investigated by several authors (Pestova *et al.*, 2005 ▶; Lilich & Djurinskii, 1956 ▶; Nicholson & Felsing, 1950 ▶; Willard & Smith, 1923 ▶) in different temperature and concentration regions. They reported the tetra­hydrate and the hexa­hydrate to be stable phases. While re-investigating the phase diagram, we found at higher temperatures the trihydrate, the tetra­hydrate at room temperature and the nona­hydrate near the eutectic temperature. The existence of the hexa­hydrate could not be confirmed.

## Structural commentary   

**Figure 1:**
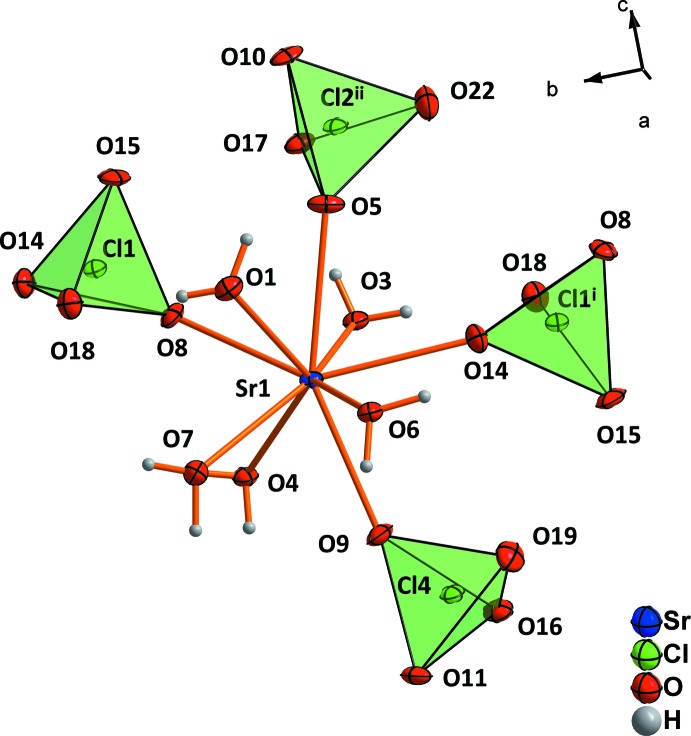
Coordination around the Sr1^2+^ cation in Sr(ClO_4_)_2_·3H_2_O. Atoms O3 and O4 as well as O6 and O1 are shared between two different Sr^2+^ cations. Displacement ellipsoids are drawn at the 50% probability level. [Symmetry codes: (i) 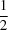 − *x*, −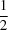 + *y*, 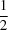 − *z*; (ii) −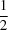 + *x*, 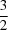 − *y*, 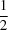 + *z*.]

**Figure 2:**
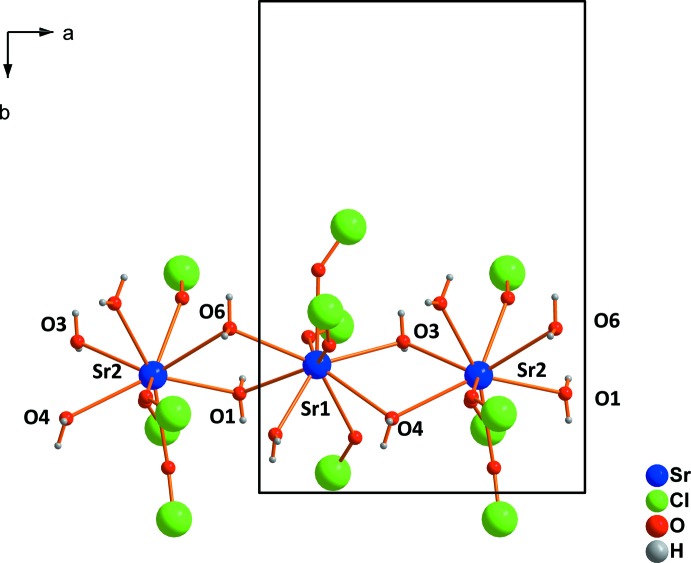
Formation of chains parallel [100] by sharing water mol­ecules in the structure of Sr(ClO_4_)_2_·3H_2_O.

**Figure 3:**
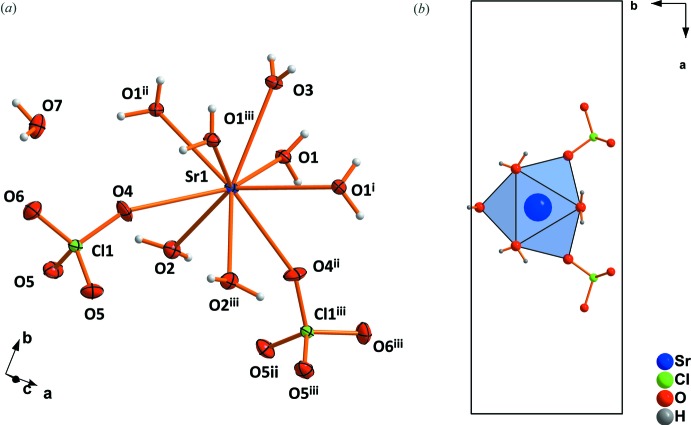
(*a*) Coordination around the Sr^2+^ cation and (*b*) the resulting coordination polyhedron in the structure of Sr(ClO_4_)_2_·9H_2_O. Displacement ellipsoids are drawn at the 50% probability level. [Symmetry codes: (i) *x*, *y*, 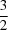 − *z*; (ii) 2 − *x*, *y*, *z*; (iii) 2 − *x*, *y*, 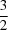 − *z*.]

The crystal structure of strontium perchlorate trihydrate contains two crystallographically distinct Sr^2+^cations. Both are coordinated by five water mol­ecules and four monodentately bonding perchlorate tetra­hedra (Fig. 1 ▶). Four of the five water mol­ecules (O1, O6 and O3, O4) share edges between two Sr^2+^ cations, resulting in chains with alternating Sr1 and Sr2 cations. The chains extend parallel to [100] (Fig. 2 ▶). The crystal structure of strontium perchlorate tetra­hydrate is similar to the trihydrate, but different to the magnesium analogue (Robertson & Bish, 2010 ▶; Solovyov, 2012 ▶) or mercury perchlor­ate tetra­hydrate (Johansson *et al.*, 1966 ▶). Two symmetry-related Sr^2+^ cations, both coordinated by five water mol­ecules and four monodentate perchlorate tetra­hedra, form dimers by sharing two water mol­ecules. In strontium perchlorate nona­hydrate, the Sr^2+^ cation occupies a single crystallographic site with site symmetry *m*2*m*. It is coordinated by seven water mol­ecules and two monodentate perchlorate tetra­hedra (point group symmetry ..*m*; Fig. 3 ▶ *a*) within a tricapped trigonal-prismatic oxygen coordination environment (Fig. 3 ▶ *b*). Thereby, the trigonal base planes are chosen such that each oxygen atom of the perchlorate anions represents a capping atom. The third cap is provided by a water oxygen atom.

## Supra­molecular features   

**Figure 4:**
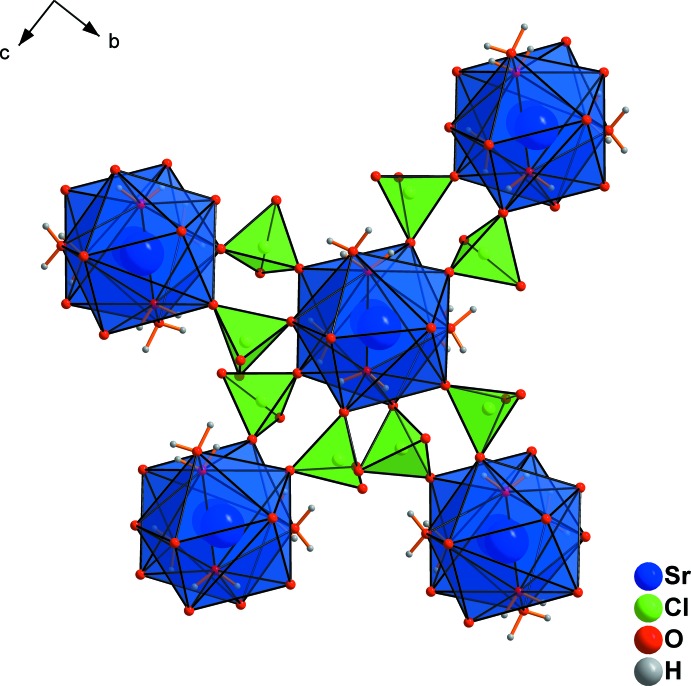
Perchlorate tetra­hedra in the structure of Sr(ClO_4_)_2_·3H_2_O linking the chains (oriented parallel to [100]) into (100) layers.

**Figure 5:**
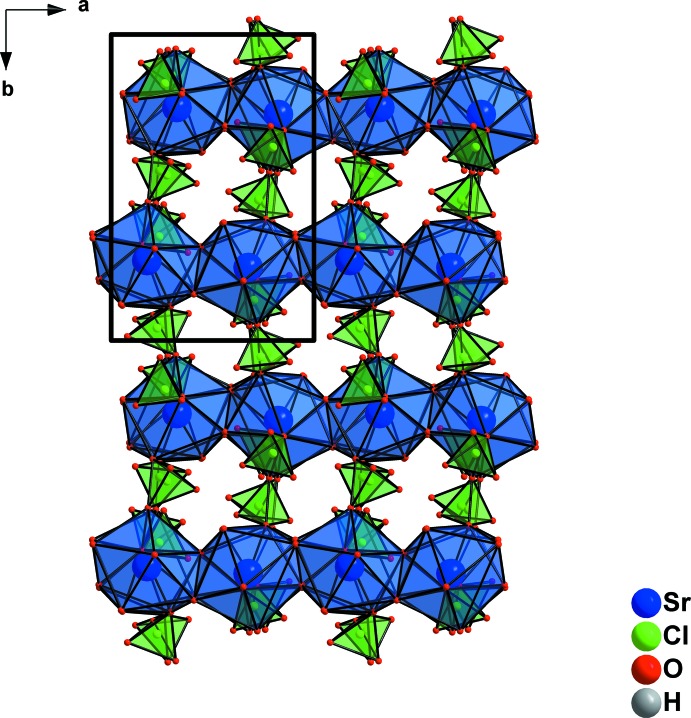
Zigzag chains parallel to [100] in the structure of Sr(ClO_4_)_2_·3H_2_O, linked by perchlorate tetra­hedra into (100) layers, as viewed along [001].

**Figure 6:**
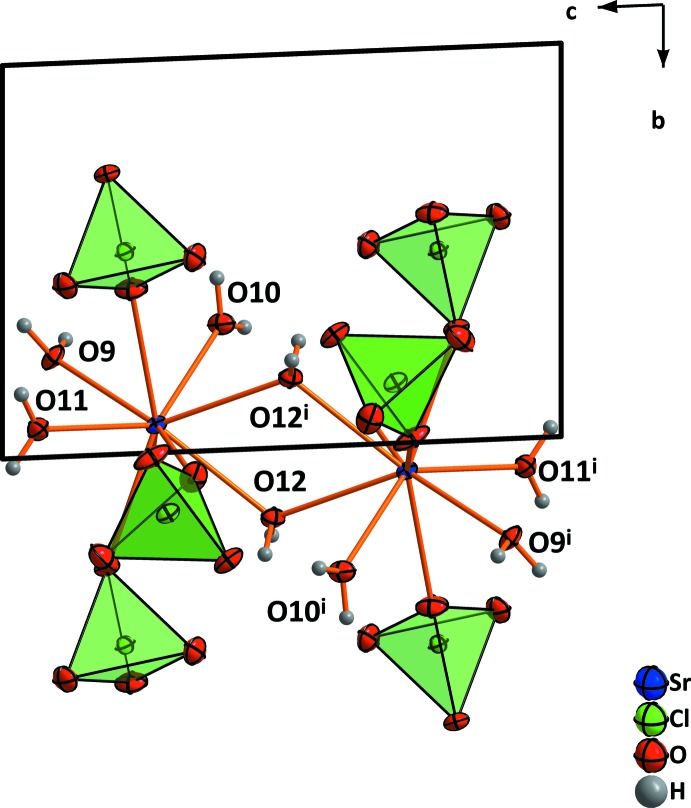
Formation of dimers in the structure of Sr(ClO_4_)_2_·4H_2_O by sharing two water mol­ecules. [Symmetry code: (i) 1 − *x*, 2 − *y*, 1 − *z*.]

**Figure 7:**
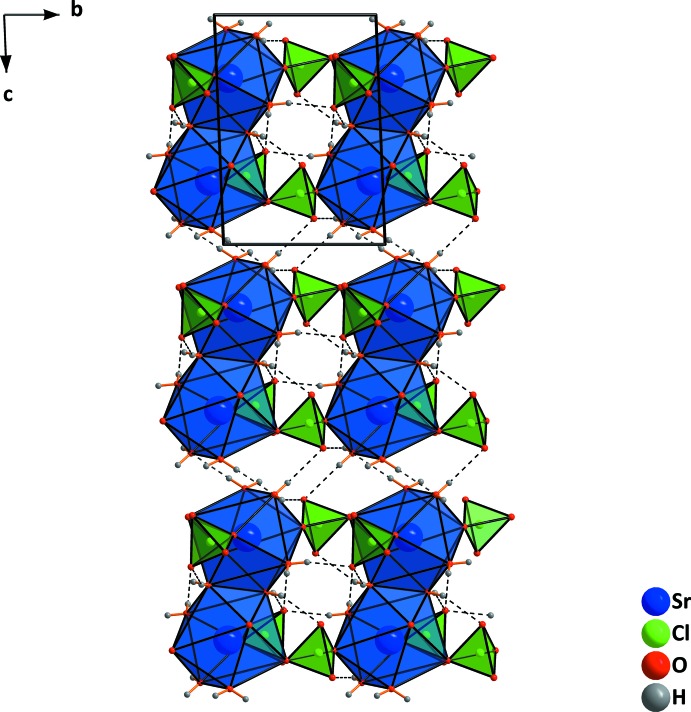
Formation of layers in the structure of Sr(ClO_4_)_2_·4H_2_O, viewed along [100].

**Figure 8:**
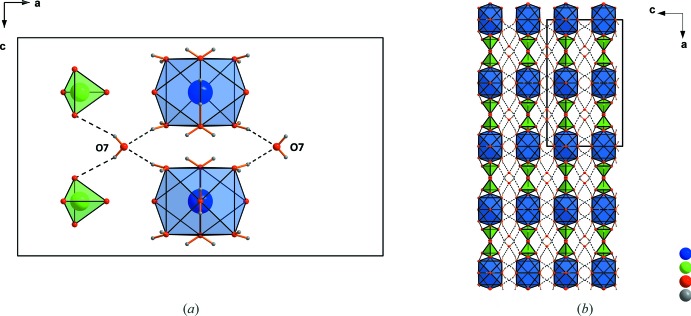
(*a*) Coordination of the lattice water mol­ecules in the structure of Sr(ClO_4_)_2_·9H_2_O by hydrogen bonds. (*b*) A larger section of the structure in the viewing direction [010]. Dashed lines indicate hydrogen bonds.

In strontium perchlorate trihydrate, chains are formed with alternating Sr^2+^ cations (Fig. 2 ▶). These zigzag chains are oriented parallel to [100] and are linked by edge-sharing with the perchlorate tetra­hedra (Fig. 4 ▶) into a layered arrangement parallel to (001), as shown in Fig. 5 ▶. Within the structure of the tetra­hydrate, each perchlorate anion coordinates to the dimeric unit of two Sr^2+^ cations (Fig. 6 ▶). At the same time, it also coordinates to another dimeric unit. Thus, each dimeric unit is connected pairwise by perchlorate anions with four others. This yields in (001) layers stacked along [001], as visualized in Fig. 7 ▶. The nona­hydrate structure contains additional lattice water mol­ecules, which are both donor and acceptor groups, resulting in a tetra­hedral arrangement of O—H⋯O hydrogen bonds. Two hydrogen bonds are formed towards the [SrO_2_(OH_2_)_7_] coordination polyhedra and two towards perchlorate tetra­hedra (Fig. 8 ▶ *a*, Table 1 ▶). The [SrO_2_(OH_2_)_7_] polyhedra additionally are linked *via* other O—H⋯O hydrogen bonds. The resulting arrangement can be seen in a larger section of the structure (Fig. 8 ▶ *b*). O—H⋯O hydrogen bonds also dominate the crystal packing in the two other structures, in each case leading to the formation of a three-dimensional network (Tables 2 ▶ and 3 ▶).

## Database survey   

For crystal structures of other *M*(ClO_4_)_2_·3H_2_O phases, see: Gallucci & Gerkin (1988 ▶; *M* = Ba); Hennings *et al.* (2014*a* ▶; Sn). For crystal structures of other *M*(ClO_4_)_2_·4H_2_O phases, see: Robertson & Bish (2010 ▶; Mg); Hennings *et al.* (2014*b* ▶; Ca); Solovyov (2012 ▶; Mg); Johansson *et al.* (1966 ▶; Hg).

## Synthesis and crystallization   

Crystals of Sr(ClO_4_)_2_·3H_2_O were used as purchased (ABCR, 98%). The isolated crystals were stored in a freezer separated and embedded in perfluorinated ether to avoid contact with humidity. Sr(ClO_4_)_2_·4H_2_O crystallized from an aqueous solution of 75.08 wt% Sr(ClO_4_)_2_ at 273 K after two days and Sr(ClO_4_)_2_·9H_2_O from an aqueous solution of 60.12 wt% Sr(ClO_4_)_2_ at 238 K after one week. For preparing these aqueous solutions, strontium perchlorate trihydrate was used. The Sr^2+^ content was analyzed per complexometric titration with EDTA. The crystals are stable in the saturated aqueous solutions over a range of at least four weeks. The samples were stored in a freezer or a cryostat at low temperatures and were separated and embedded in perfluorinated ether for X-ray analysis.

## Refinement   

Crystal data, data collection and structure refinement details are summarized in Table 4 ▶. The H atoms of each structure were placed in the positions indicated by difference Fourier maps. For Sr(ClO_4_)_2_·3H_2_O and Sr(ClO_4_)_2_·4H_2_O distance restraints were applied for all water mol­ecules, with O—H and H—H distance restraints of 0.84 (1) and 1.4 (1) Å, respectively. For Sr(ClO_4_)_2_·9H_2_O *U*_iso_ values were set at 1.2*U*_eq_(O) using a riding model approximation. Distance restraints were applied for that structure for all water mol­ecules, with O—H and H—H distance restraints of 0.84 (1) and 1.4 (1) Å, respectively.

## Supplementary Material

Crystal structure: contains datablock(s) SrClO4_3H2O_100K, SrClO4_4H2O_150K, SrClO4_9H2O_100K. DOI: 10.1107/S1600536814024726/wm5080sup1.cif

Structure factors: contains datablock(s) SrClO4_3H2O_100K. DOI: 10.1107/S1600536814024726/wm5080SrClO4_3H2O_100Ksup2.hkl

Structure factors: contains datablock(s) SrClO4_4H2O_150K. DOI: 10.1107/S1600536814024726/wm5080SrClO4_4H2O_150Ksup3.hkl

Structure factors: contains datablock(s) SrClO4_9H2O_100K. DOI: 10.1107/S1600536814024726/wm5080SrClO4_9H2O_100Ksup4.hkl

Supporting information file. DOI: 10.1107/S1600536814024726/wm5080SrClO4_3H2O_100Ksup5.cml

Supporting information file. DOI: 10.1107/S1600536814024726/wm5080SrClO4_4H2O_150Ksup6.cml

Supporting information file. DOI: 10.1107/S1600536814024726/wm5080SrClO4_9H2O_100Ksup7.cml

CCDC references: 1033590, 1033589, 1033588

Additional supporting information:  crystallographic information; 3D view; checkCIF report

## Figures and Tables

**Table 1:** Hydrogen-bond geometry (, ) for Sr(ClO_4_)_2_9H_2_O

*D*H*A*	*D*H	H*A*	*D* *A*	*D*H*A*
O1H1*B*O7^i^	0.84(1)	2.02(2)	2.844(4)	169(5)
O1H1*A*O4	0.84(1)	1.98(1)	2.811(4)	170(5)
O2H2*A*O1^ii^	0.84(1)	1.99(2)	2.780(4)	156(5)
O3H3*A*O2^i^	0.84(1)	2.05(3)	2.851(5)	158(7)
O4H4*A*O6^iii^	0.84(1)	2.62(3)	3.337(2)	144(4)
O4H4*A*O7^iv^	0.84(1)	2.39(3)	3.041(4)	135(4)

Symmetry codes: (i) 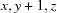; (ii) 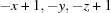; (iii) 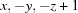; (iv) 
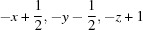
.

**Table 2:** Hydrogen-bond geometry (, ) for Sr(ClO_4_)_2_3H_2_O

*D*H*A*	*D*H	H*A*	*D* *A*	*D*H*A*
O1H1*A*O5^i^	0.84(1)	2.13(4)	2.683(4)	123(4)
O1H1*B*O18^i^	0.84(1)	2.07(2)	2.858(4)	158(4)
O2H2*B*O16^ii^	0.84(1)	2.10(1)	2.923(4)	169(4)
O2H2*A*O16	0.84(1)	2.17(2)	2.992(4)	167(7)
O3H3*A*O18^iii^	0.84(1)	1.96(1)	2.793(4)	172(4)
O3H3*B*O17^iv^	0.84(1)	2.06(2)	2.857(4)	159(4)
O4H4*B*O21^v^	0.84(1)	2.15(2)	2.953(4)	161(5)
O4H4*A*O22^vi^	0.84(1)	2.42(2)	3.173(4)	150(4)
O6H6*A*O14^iii^	0.84(7)	2.56(7)	3.069(4)	121(5)
O6H6*A*O19^vii^	0.84(7)	2.19(7)	2.964(4)	155(6)
O6H6*B*O17^viii^	0.92(6)	2.09(6)	2.920(4)	150(5)
O7H7*A*O20^ix^	0.84(1)	2.31(4)	3.044(4)	146(7)
O7H7*A*O22^vi^	0.84(1)	2.44(7)	2.902(4)	116(6)
O7H7*B*O17^viii^	0.84(1)	2.48(5)	2.916(4)	114(4)
O7H7*B*O20^v^	0.84(1)	2.25(2)	3.071(4)	167(5)

Symmetry codes: (i) 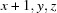; (ii) 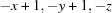; (iii) 
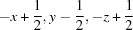
; (iv) 
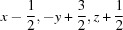
; (v) 
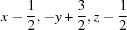
; (vi) 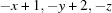; (vii) 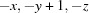; (viii) 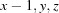; (ix) 
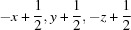
.

**Table 3:** Hydrogen-bond geometry (, ) for Sr(ClO_4_)_2_4H_2_O

*D*H*A*	*D*H	H*A*	*D* *A*	*D*H*A*
O9H9*A*O8^i^	0.84(1)	2.15(2)	2.966(3)	164(4)
O9H9*B*O8^ii^	0.84(1)	2.18(2)	2.986(3)	161(5)
O10H10*B*O4^iii^	0.84(1)	2.04(2)	2.858(3)	165(6)
O10H10*A*O4^iv^	0.84(1)	2.17(2)	2.967(3)	157(5)
O11H11*B*O9^v^	0.84(1)	1.99(2)	2.809(3)	164(4)
O11H11*A*O8	0.84(1)	2.38(3)	3.093(3)	143(5)
O12H12*A*O7^vi^	0.84(1)	2.23(2)	2.986(3)	150(4)
O12H12*A*O10^vii^	0.84(1)	2.31(4)	2.820(3)	120(3)
O12H12*B*O4	0.84(1)	2.06(2)	2.875(3)	164(5)

Symmetry codes: (i) 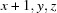; (ii) 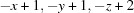; (iii) 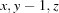; (iv) 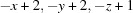; (v) 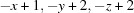; (vi) 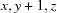; (vii) 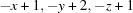.

**Table 4:** Experimental details

	Sr(ClO_4_)_2_3H_2_O	Sr(ClO_4_)_2_4H_2_O	Sr(ClO_4_)_2_9H_2_O
Crystal data			
*M*_r_	340.57	358.58	448.66
Crystal system, space group	Monoclinic, *P*2_1_/*n*	Triclinic, *P* 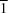	Orthorhombic, *C* *m* *c* *m*
Temperature (K)	100	150	100
*a*, *b*, *c* ()	8.9787(6), 13.4870(12), 14.7875(10)	7.1571(6), 7.3942(6), 10.0231(9)	18.7808(15), 6.860(3), 11.1884(16)
, , ()	90, 95.448(5), 90	86.674(7), 86.291(7), 72.027(6)	90, 90, 90
*V* (^3^)	1782.6(2)	503.09(8)	1441.5(7)
*Z*	8	2	4
Radiation type	Mo *K*	Mo *K*	Mo *K*
(mm^1^)	6.70	5.94	4.20
Crystal size (mm)	0.45 0.34 0.23	0.33 0.25 0.16	0.20 0.11 0.05

Data collection			
Diffractometer	Stoe IPDS 2T	Stoe IPDS 2T	Stoe IPDS 2T
Absorption correction	Integration (Coppens, 1970 ▶)	Integration (Coppens, 1970 ▶)	Integration (Coppens, 1970 ▶)
*T*_min_, *T*_max_	0.081, 0.212	0.187, 0.383	0.015, 0.085
No. of measured, independent and observed [*I* > 2(*I*)] reflections	50555, 4941, 3337	10691, 2818, 2650	6877, 1087, 993
*R*_int_	0.125	0.065	0.020
(sin /)_max_ (^1^)	0.650	0.695	0.693

Refinement			
*R*[*F* ^2^ > 2(*F* ^2^)], *wR*(*F* ^2^), *S*	0.024, 0.046, 1.09	0.028, 0.076, 1.10	0.048, 0.134, 1.16
No. of reflections	4087	2795	1087
No. of parameters	297	169	70
No. of restraints	15	12	6
H-atom treatment	H atoms treated by a mixture of independent and constrained refinement	All H-atom parameters refined	Only H-atom coordinates refined
_max_, _min_ (e ^3^)	0.56, 0.63	0.83, 1.15	1.27, 2.26

Computer programs: *X-AREA* and *X-RED* (Stoe Cie, 2009 ▶), *SHELXS97* and *SHELXL2012* (Sheldrick, 2008 ▶), *DIAMOND* (Brandenburg, 2006 ▶) and *publCIF* (Westrip, 2010 ▶).

## supplementary crystallographic information

### Crystal data

**Table:**

[Sr(ClO_4_)_2_(H_2_O)_7_]·2H_2_O	*D*_x_ = 2.067 Mg m^−^^3^
*M**_r_* = 448.66	Mo *K*α radiation, λ = 0.71073 Å
Orthorhombic, *C**m**c**m*	Cell parameters from 5894 reflections
*a* = 18.7808 (15) Å	θ = 8.3–29.6°
*b* = 6.860 (3) Å	µ = 4.20 mm^−^^1^
*c* = 11.1884 (16) Å	*T* = 100 K
*V* = 1441.5 (7) Å^3^	Prism, colourless
*Z* = 4	0.2 × 0.11 × 0.05 mm
*F*(000) = 904	

### Data collection

**Table:**

Stoe IPDS 2T diffractometer	1087 independent reflections
Radiation source: fine-focus sealed tube	993 reflections with *I* > 2σ(*I*)
Detector resolution: 6.67 pixels mm^-1^	*R*_int_ = 0.020
rotation method scans	θ_max_ = 29.5°, θ_min_ = 3.2°
Absorption correction: integration (Coppens, 1970)	*h* = −26→26
*T*_min_ = 0.015, *T*_max_ = 0.085	*k* = −9→9
6877 measured reflections	*l* = −15→15

### Refinement

**Table:**

Refinement on *F*^2^	6 restraints
Least-squares matrix: full	Hydrogen site location: difference Fourier map
*R*[*F*^2^ > 2σ(*F*^2^)] = 0.048	Only H-atom coordinates refined
*wR*(*F*^2^) = 0.134	*w* = 1/[σ^2^(*F*_o_^2^) + (0.1*P*)^2^] where *P* = (*F*_o_^2^ + 2*F*_c_^2^)/3
*S* = 1.16	(Δ/σ)_max_ < 0.001
1087 reflections	Δρ_max_ = 1.27 e Å^−^^3^
70 parameters	Δρ_min_ = −2.26 e Å^−^^3^

### Special details

**Table:**

Geometry. All esds (except the esd in the dihedral angle between two l.s. planes) are estimated using the full covariance matrix. The cell esds are taken into account individually in the estimation of esds in distances, angles and torsion angles; correlations between esds in cell parameters are only used when they are defined by crystal symmetry. An approximate (isotropic) treatment of cell esds is used for estimating esds involving l.s. planes.

### Fractional atomic coordinates and isotropic or equivalent isotropic displacement parameters (Å^2^)

**Table:**

	*x*	*y*	*z*	*U*_iso_*/*U*_eq_	
Sr1	0.5000	0.05870 (8)	0.2500	0.0096 (2)	
Cl1	0.32953 (5)	−0.30996 (15)	0.2500	0.0113 (3)	
O7	0.34391 (14)	−0.4247 (4)	0.3560 (2)	0.0177 (5)	
O4	0.29063 (18)	0.0000	0.5000	0.0203 (7)	
H4A	0.263 (2)	0.041 (7)	0.553 (3)	0.024*	
O2	0.5000	−0.2257 (5)	0.4061 (3)	0.0156 (7)	
H2A	0.5369 (16)	−0.215 (6)	0.448 (4)	0.019*	
O1	0.40632 (13)	0.2050 (4)	0.4020 (2)	0.0147 (5)	
H1A	0.3693 (15)	0.146 (6)	0.424 (4)	0.018*	
H1B	0.392 (2)	0.315 (3)	0.380 (5)	0.018*	
O5	0.3748 (2)	−0.1397 (5)	0.2500	0.0211 (8)	
O6	0.2561 (2)	−0.2480 (6)	0.2500	0.0209 (8)	
O3	0.5000	0.4459 (6)	0.2500	0.0141 (9)	
H3A	0.5000	0.519 (9)	0.310 (4)	0.017*	

### Atomic displacement parameters (Å^2^)

**Table:**

	*U*^11^	*U*^22^	*U*^33^	*U*^12^	*U*^13^	*U*^23^
Sr1	0.0111 (3)	0.0142 (3)	0.0035 (3)	0.000	0.000	0.000
Cl1	0.0105 (5)	0.0152 (5)	0.0083 (5)	−0.0003 (3)	0.000	0.000
O7	0.0221 (11)	0.0230 (11)	0.0079 (11)	0.0032 (10)	0.0004 (9)	0.0033 (8)
O4	0.0117 (15)	0.0346 (18)	0.0147 (17)	0.000	0.000	0.0023 (16)
O2	0.0144 (14)	0.0219 (15)	0.0106 (16)	0.000	0.000	0.0009 (13)
O1	0.0143 (10)	0.0202 (10)	0.0095 (10)	−0.0006 (9)	0.0009 (8)	−0.0001 (9)
O5	0.0208 (17)	0.0181 (16)	0.0243 (19)	−0.0067 (14)	0.000	0.000
O6	0.0096 (15)	0.0243 (17)	0.029 (2)	0.0032 (12)	0.000	0.000
O3	0.021 (2)	0.0122 (19)	0.009 (2)	0.000	0.000	0.000

### Geometric parameters (Å, º)

**Table:**

Sr1—O2	2.619 (4)	Sr1—O5	2.716 (4)
Sr1—O2^i^	2.619 (4)	Sr1—O5^ii^	2.716 (4)
Sr1—O1^ii^	2.645 (2)	Cl1—O6	1.443 (4)
Sr1—O1^i^	2.645 (2)	Cl1—O5	1.445 (4)
Sr1—O1^iii^	2.645 (2)	Cl1—O7	1.449 (3)
Sr1—O1	2.645 (2)	Cl1—O7^i^	1.449 (3)
Sr1—O3	2.656 (5)		
			
O2—Sr1—O2^i^	83.68 (16)	O2^i^—Sr1—O5	68.08 (7)
O2—Sr1—O1^ii^	81.60 (8)	O1^ii^—Sr1—O5	139.99 (5)
O2^i^—Sr1—O1^ii^	135.37 (6)	O1^i^—Sr1—O5	67.32 (8)
O2—Sr1—O1^i^	135.37 (6)	O1^iii^—Sr1—O5	139.99 (5)
O2^i^—Sr1—O1^i^	81.60 (8)	O1—Sr1—O5	67.33 (8)
O1^ii^—Sr1—O1^i^	135.38 (11)	O3—Sr1—O5	120.07 (8)
O2—Sr1—O1^iii^	135.37 (6)	O2—Sr1—O5^ii^	68.08 (7)
O2^i^—Sr1—O1^iii^	81.60 (8)	O2^i^—Sr1—O5^ii^	68.08 (7)
O1^ii^—Sr1—O1^iii^	80.01 (11)	O1^ii^—Sr1—O5^ii^	67.32 (8)
O1^i^—Sr1—O1^iii^	83.41 (11)	O1^i^—Sr1—O5^ii^	139.99 (5)
O2—Sr1—O1	81.60 (8)	O1^iii^—Sr1—O5^ii^	67.32 (8)
O2^i^—Sr1—O1	135.37 (6)	O1—Sr1—O5^ii^	139.99 (5)
O1^ii^—Sr1—O1	83.41 (11)	O3—Sr1—O5^ii^	120.07 (8)
O1^i^—Sr1—O1	80.01 (11)	O5—Sr1—O5^ii^	119.85 (17)
O1^iii^—Sr1—O1	135.38 (11)	O6—Cl1—O5	108.9 (2)
O2—Sr1—O3	138.16 (8)	O6—Cl1—O7	109.77 (14)
O2^i^—Sr1—O3	138.16 (8)	O5—Cl1—O7	109.23 (14)
O1^ii^—Sr1—O3	67.69 (6)	O6—Cl1—O7^i^	109.77 (14)
O1^i^—Sr1—O3	67.69 (6)	O5—Cl1—O7^i^	109.24 (14)
O1^iii^—Sr1—O3	67.69 (6)	O7—Cl1—O7^i^	109.9 (2)
O1—Sr1—O3	67.69 (6)	Cl1—O5—Sr1	156.2 (2)
O2—Sr1—O5	68.08 (7)		

Symmetry codes: (i) *x*, *y*, −*z*+1/2; (ii) −*x*+1, *y*, *z*; (iii) −*x*+1, *y*, −*z*+1/2.

### Hydrogen-bond geometry (Å, º)

**Table:**

*D*—H···*A*	*D*—H	H···*A*	*D*···*A*	*D*—H···*A*
O1—H1*B*···O7^iv^	0.84 (1)	2.02 (2)	2.844 (4)	169 (5)
O1—H1*A*···O4	0.84 (1)	1.98 (1)	2.811 (4)	170 (5)
O2—H2*A*···O1^v^	0.84 (1)	1.99 (2)	2.780 (4)	156 (5)
O3—H3*A*···O2^iv^	0.84 (1)	2.05 (3)	2.851 (5)	158 (7)
O4—H4*A*···O6^vi^	0.84 (1)	2.62 (3)	3.337 (2)	144 (4)
O4—H4*A*···O7^vii^	0.84 (1)	2.39 (3)	3.041 (4)	135 (4)

Symmetry codes: (iv) *x*, *y*+1, *z*; (v) −*x*+1, −*y*, −*z*+1; (vi) *x*, −*y*, −*z*+1; (vii) −*x*+1/2, −*y*−1/2, −*z*+1.
